# Catalyzing NTD gender and equity research: A call for papers

**DOI:** 10.1371/journal.pntd.0006681

**Published:** 2018-10-18

**Authors:** Arianna Rubin Means, Alison Krentel, Sally Theobald, Laura Dean, Pamela Sabina Mbabazi, Thoko Elphick-Pooley, Fiona M. Fleming, Julie Jacobson, Sarah Simpson, Camilla Ducker

**Affiliations:** 1 Department of Global Health, University of Washington, Seattle, Washington, United States of America; 2 Bruyère Research Institute and School of Epidemiology and Public Health, University of Ottawa, Ottowa, Canada; 3 Department of International Public Health, Liverpool School of Tropical Medicine, Liverpool, United Kingdom; 4 Department of Control of Neglected Tropical Diseases, World Health Organization, Geneva, Switzerland; 5 Uniting to Combat NTDs Support Centre, Brighton and Hove, United Kingdom; 6 Schistosomiasis Control Initiative, School of Public Health, Imperial College London, United Kingdom; 7 EquiACT, Lyon, France; Yale School of Public Health, UNITED STATES

Neglected tropical disease (NTD) programs are among the largest community-based public health interventions in existence. More than 1 billion people were reached with preventive chemotherapy for at least one NTD in 2016 alone [1]. The significance of the NTD program is predicated upon the opportunity to treat disease, prevent morbidity and disability, and ultimately interrupt infection transmission among large populations. Additionally, the untapped potential of these programs lies in the possibility of reaching hard-to-reach individuals with additional health services that may otherwise be inaccessible to them due to a myriad of geographic, social, or cultural obstacles.

NTDs disproportionality affect socially and economically marginalized populations globally, and by virtue, NTD programs can provide invaluable healthcare access opportunities for individuals or groups of individuals who are otherwise disenfranchised or isolated. There is a need to build on existing NTD platforms to ensure that these opportunities are available to all individuals regardless of their geographic and social positioning. Accordingly, coverage of NTD interventions has also been proposed as an “equity” tracer within the 2030 Sustainable Development Goals (SDGs) [2].

As also emphasized in the 2030 Agenda for Sustainable Development, gender roles and relations—and the ways in which they interact with other intersecting inequities—are often at the heart of inadequate healthcare delivery or access. For NTDs, sex and gender roles can have a profound effect on healthcare access, health outcomes, and caregiver responsibilities [3]. There is evidence that NTDs influence adverse birth outcomes for pregnant women and women of reproductive age, resulting in chronic anemia, premature birth, and even increased risk of maternal mortality [4, 5]. Likewise, women infected with some NTDs may face a disproportionate risk of acquiring sexually transmitted infections such as HIV [6], or social exclusion and stigma if they develop NTD-associated morbidities and disability [7, 8]. Risk of NTD infection may also be gendered, often based upon social differentiation of occupational and household tasks [9, 10]. Importantly, gender also intersects with other axes of inequity such as ethnicity, socioeconomic status, occupation status, age, sexuality, (dis)ability, or religion, and there is increasing interest in gender and intersectionality analysis to address key global health priority issues [11]. For example, gender norms can affect the ability of women to participate as community volunteers in preventive chemotherapy programs due to the influence of existing cultural expectations and social hierarchies, affecting their occupational and social engagement opportunities [12]. These intersections compound risk of NTD acquisition, experiences of morbidity, disability and illness, poverty, and access to high-quality care within different contexts, further aggravating inequities.

Despite the known mediating role of gender in influencing health and social well-being, there is a dearth of research regarding the gender equity of NTD programs. There is also minimal understanding of the influence of sex, gender, and other intersecting inequities on specific health and development outcomes, including the functioning of NTD programs and the broader health systems they link with [13]. A deeper understanding of how and why sex and gender influence factors such as NTD acquisition risk, disease experience, morbidity and disability consequences, or programmatic delivery and access is critical not only for candid introspection as an NTD community but also in designing more effective and equitable programs that leave no one behind.

The Uniting to Combat NTDs Equity Working Group, in partnership with *PLOS Neglected Tropical Diseases*, is inviting manuscripts for a special journal supplement on gender and NTDs. The purpose of the supplement is to catalyze NTD research that addresses the issue of gender equity within research, organizational, policy, and practice activities at different levels of the health system. Ultimately, it is our hope that the supplement will promote recognition of the necessity of mainstreaming gender and sex research within NTD studies and programs, showcase emerging research in this area, and spark new and innovative collaborations.

We invite papers from single or multiple contexts that span the translational research spectrum and are of any methodological underpinning. We also encourage reports from initiatives that have attempted to address gender, sex, and associated equity or inequities in NTD research or programs that might influence future research and operational practices. Specific papers that are relevant for inclusion in this supplement include:

1. Papers that utilize a gender lens to assess the risk of disease exposure and the intersection with social and structural determinants of NTD infection and morbidity. These determinants may include water and sanitation, housing and clustering, environmental factors, migration, disasters and conflicts, sociocultural factors, or poverty, to name a few [14].
2. Papers that address sex or gender-associated health or social outcomes of NTDs, including the ways in which gender and other axes of inequity shape individual or household-level indicators of well-being relevant to NTD programs.
3. Papers that address gender at all levels of the NTD workforce and/or the leadership of NTD-oriented programs and organizations. This may include a focus on community drug distributors, research study staff, NTD program leadership, regional and global agencies, or other relevant workforce participants and the strengths and weaknesses of associated NTD programs.
4. Papers that address gender and equity in NTD program delivery including mass drug administration coverage, utilization of water and sanitation resources, vector control interventions, and access to disease management services for morbidity, disability, or mental health services.
5. Papers that address strategies to tailor NTD outreach programs to meet the gender-specific needs and priorities of specific targeted populations.

Given the scale and scope of NTD programs, a strengthened and renewed focus on equity and gender mainstreaming could have a profound effect on public health delivery for marginalized populations globally, particularly if these delivery platforms are utilized for other health interventions. Controlling and eliminating NTDs may be a direct strategy for strengthening gender and social equity in endemic areas, a critical element of universal health coverage [15]. Likewise, addressing gender and social inequities could have societal effects that alleviate some of the conditions favorable to the spread of NTDs. Therefore, we hope that this supplement not only emphasizes the deep roots of NTDs in social inequities but also catalyzes rigorous scientific research that broadly contributes to building a healthier and more just world.

All supplement submissions are due by 1 June 2019. Please see the Submission Guidelines (link:https://journals.plos.org/plosntds/s/submission-guidelines) for more details.
